# Age-Specific Transcriptomic Signatures for Classification of Progression-Free Survival Outcomes in Luminal A Breast Cancer: An Integrative Machine Learning Approach

**DOI:** 10.3390/biology15141160

**Published:** 2026-07-15

**Authors:** Mehmet Kivrak, Ihsan Nalkiran, Hatice Sevim Nalkiran

**Affiliations:** 1 Department of Biostatistics and Medical Informatics, Faculty of Medicine, Recep Tayyip Erdogan University, 53020 Rize, Türkiye; mehmet.kivrak@erdogan.edu.tr; 2 Department of Medical Biology, Faculty of Medicine, Recep Tayyip Erdogan University, 53020 Rize, Türkiye; hatice.sevim@erdogan.edu.tr

**Keywords:** Luminal A breast cancer, progression-free survival, transcriptomic signatures, machine learning, recurrence

## Abstract

**Simple Summary:**

Progression-free survival is an important indicator of disease outcome in Luminal A breast cancer (BC), but the molecular alterations associated with recurrence may vary across age-defined patient subgroups. In this study, we integrated transcriptomic and clinical data to identify recurrence-associated molecular biomarkers and develop machine learning-based prediction models. Several genes, including *KMT2D*, *RFNG*, *IGF1*, and *CDKN2C*, showed consistent recurrence-associated expression patterns across patient subgroups, whereas *ATM*, *RPTOR*, and *RICTOR* displayed subgroup-dependent expression profiles. We also identified age, Nottingham Prognostic Index (NPI), and tumor size as the most important clinical predictors of recurrence. Integrating transcriptomic biomarkers with these clinical variables improved recurrence classification compared with models based only on clinicopathological variables. These findings highlight the potential value of combining molecular and clinical information for recurrence risk assessment and identify candidate biomarkers for future validation in Luminal A BC.

**Abstract:**

Progression-free survival (PFS) is an important clinical endpoint in Luminal A breast cancer (BC), yet the molecular determinants of recurrence remain incompletely understood. This study aimed to identify recurrence-associated transcriptomic alterations across age-defined patient subgroups and evaluate their predictive utility using machine learning (ML). Gene expression profiles and clinical data from the METABRIC cohort were analyzed in premenopausal, postmenopausal non-geriatric, and geriatric patients with Luminal A BC. Differential expression analysis identified 32 significantly dysregulated genes, of which 15 genes with |log_2_FC| ≥ 2 were selected for detailed characterization. Among these, *KMT2D*, *RFNG*, *IGF1*, and *CDKN2C* exhibited consistent recurrence-associated expression patterns across patient subgroups, whereas *ATM*, *RPTOR*, and *RICTOR* displayed subgroup-dependent expression profiles. Feature selection analysis identified *KMT2D* as the most influential molecular predictor, while age, NPI, and tumor size were the most important clinical variables. The integrated clinicopathological–transcriptomic XGBoost model achieved the best predictive performance (AUC = 0.71; accuracy = 0.78) and outperformed baseline models based only on clinicopathological variables. Sensitivity analysis and independent external validation supported the robustness of the principal candidate biomarkers. These findings provide preliminary evidence supporting the investigation of transcriptomic biomarkers in combination with clinical variables for recurrence classification in Luminal A BC. However, further validation in independent cohorts is needed before their potential utility can be fully assessed.

## 1. Introduction

Breast cancer (BC) is one of the most frequently diagnosed malignancies in women and remains a leading cause of cancer-related mortality worldwide. The biological heterogeneity of breast tumors and their variable responses to treatment make accurate prognostic prediction critically important [1,2]. BC is a heterogeneous disease driven by complex genetic and environmental interactions, resulting in distinct molecular subtypes with diverse clinical outcomes. Advances in screening technologies have also increased the detection of pre-neoplastic breast lesions with the potential to progress to invasive carcinoma [3,4]. Early detection of breast lesions and accurate prognostic assessment are critical for improving treatment outcomes. However, the limited sensitivity and specificity of mammography may restrict its effectiveness in early diagnosis [5,6]. In hormone receptor-positive (HR+) BC, the risk of recurrence may persist for many years after the initial diagnosis [7]. In BC, estrogen receptor, progesterone receptor, and human epidermal growth factor receptor 2 (HER2) are key biomarkers used for molecular subtype classification and treatment decision-making [8,9,10]. These findings highlight the potential value of developing improved prognostic approaches for recurrence risk assessment in Luminal A BC.

Numerous prognostic models have been developed for breast cancer; however, only a limited number have been extensively validated across different clinical settings. More importantly, the performance of these models remains suboptimal in independent populations, particularly among high-risk patients and at the extremes of age [11]. Most of these models are primarily based on clinicopathological variables and may not fully capture the molecular heterogeneity of tumors. Moreover, despite substantial efforts toward early detection of recurrent disease, only a small proportion of recurrences are identified during the asymptomatic stage [12,13]. In recent years, artificial intelligence (AI)-based approaches have gained increasing attention in clinical cancer research and have shown promising results in prognostic modeling [14,15]. Machine learning (ML) techniques, in particular, facilitate the extraction of clinically relevant patterns from large-scale, high-dimensional biological datasets, thereby supporting clinical decision-making [16,17]. As an objective and reproducible analytical framework, ML enables the comprehensive evaluation of multiple quantitative variables and has become an important tool for enhancing diagnostic and prognostic performance [18]. Recent advances in biomarker discovery, omics technologies, and prognostic modeling have significantly enhanced the prediction of treatment resistance and disease recurrence [19,20,21,22]. Similar transcriptome-based computational approaches have successfully identified disease-associated molecular targets and signaling pathways across multiple cancer types, including small cell lung cancer [23]. In particular, gene expression profiling has contributed substantially to prognostic assessment by enabling the stratification of BC patients into distinct risk groups [24,25]. With advances in sequencing technologies and reduced costs, high-dimensional molecular datasets have become increasingly accessible, enabling artificial intelligence-based approaches to integrate molecular and clinical information into robust prognostic models [26,27,28,29]. Collectively, these integrated approaches enhance our understanding of tumor biology and provide a robust framework for the development of more accurate prognostic models [30].

In a recent study using the same Molecular Taxonomy of Breast Cancer International Consortium (METABRIC) cohort, we evaluated overall survival (OS) in patients with Luminal A BC and demonstrated that an XGBoost-based model integrating genetic markers, including *ATM* and *HERC2*, with clinical variables could predict OS with high accuracy [31]. The present study extends this approach to progression-free survival (PFS) and aims to investigate recurrence-associated transcriptomic signatures across age-defined patient subgroups and their relationship with PFS outcomes.

Although OS is considered the gold standard endpoint for evaluating treatment efficacy in oncology, PFS is frequently used as a primary endpoint in clinical studies due to the longer follow-up required to assess OS outcomes [32,33]. Notably, patients with HR+ BC remain at risk of recurrence for many years following the initial diagnosis [7]. This highlights the need for improved prognostic models capable of predicting disease progression and recurrence risk.

ML approaches may contribute to the development of personalized progression risk prediction [34]. In addition, ML has attracted increasing interest as a strategy for improving predictive performance through the integration of clinical, molecular, and pathological data [35,36]. Considering these factors, this study aimed to investigate recurrence-associated transcriptomic alterations across age-defined patient subgroups and their association with PFS outcomes in Luminal A BC. To achieve this, differentially expressed genes (DEGs) and clinical variables were evaluated across age-defined subgroups within METABRIC cohort, including premenopausal, postmenopausal non-geriatric, and geriatric patients, and integrated into ML models for recurrence classification. This approach was designed to identify recurrence-associated transcriptomic biomarkers across age-defined patient subgroups and establish a predictive framework that may improve recurrence-related risk stratification in Luminal A BC.

## 2. Materials and Methods

### 2.1. Bioinformatic Processing of Microarray-Based Gene Expression Data

**Figure 1:**
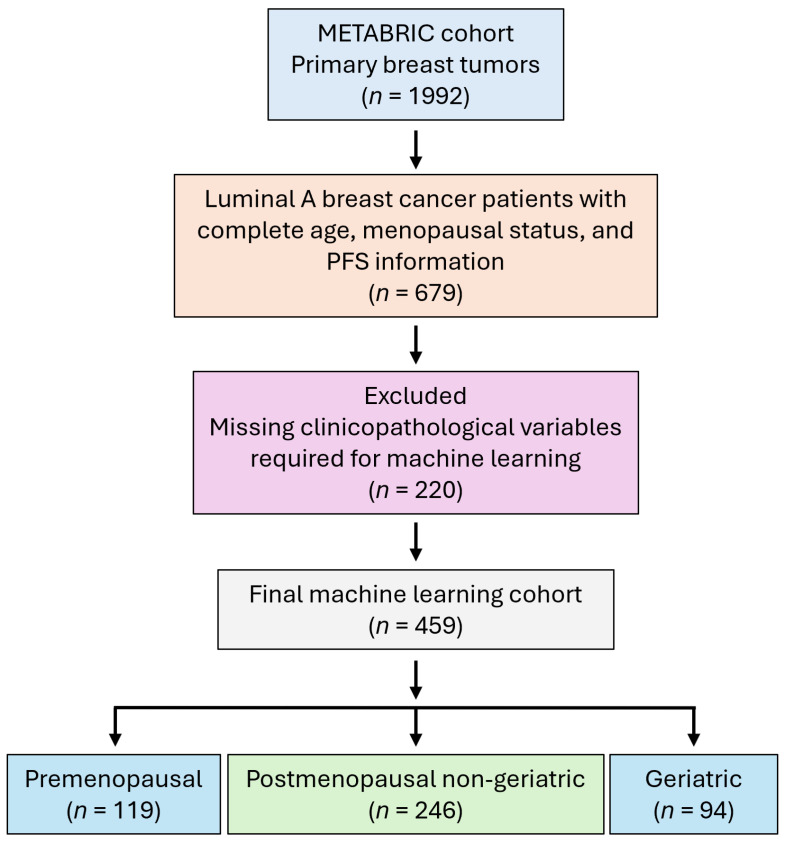
Study flow diagram illustrating patient selection and cohort construction from the METABRIC dataset.

This study used data from the METABRIC cohort, originally reported by Curtis et al. [37]. The METABRIC dataset provides genome-wide expression profiling data for 1992 primary breast tumor samples generated on the Illumina HumanHT-12 v3 Expression BeadChip platform. Microarray expression data (Illumina, San Diego, CA, USA) and corresponding clinical annotations were obtained from cBioPortal (https://www.cbioportal.org/, accessed on 1 June 2025). The METABRIC gene expression data available through cBioPortal consist of processed and normalized microarray expression values generated using the Illumina HumanHT-12 v3 platform. Therefore, raw microarray intensity files were not reprocessed in the present study. Additional preprocessing consisted of gene annotation matching, filtering of low-variance genes, and quality control prior to downstream transcriptomic analyses. For the present study, PFS was defined as a composite endpoint including disease recurrence, locoregional progression, distant metastasis, second primary tumor occurrence, or death from any cause. Patients classified as Luminal A with complete information on age, menopausal status, and PFS outcomes were eligible for inclusion. Menopausal status was obtained from the original METABRIC clinical annotations, and patients were initially classified as premenopausal or postmenopausal according to the original METABRIC classification. The postmenopausal group was subsequently stratified according to age into postmenopausal non-geriatric (<70 years) and geriatric (≥70 years) groups. After excluding cases with incomplete demographic or PFS-related information, 679 patients remained for age-stratified transcriptomic analyses. The overall patient selection process and cohort construction are illustrated in Figure 1. After further exclusion of cases with missing clinicopathological variables required for machine learning, the final predictive modeling cohort consisted of 459 patients. The distribution of patients according to age-defined subgroups and recurrence status is summarized in Table 1.

### 2.2. Data Preprocessing

Gene expression preprocessing was conducted in R software (version 4.2.3) using the dplyr and limma packages. Gene identifiers were matched to reference annotations, and genes with low variability across samples were removed using interquartile range filtering. Log_2_ transformation and normalization were subsequently applied to reduce technical variability and improve comparability across samples. Additional exclusion criteria were applied to remove cases lacking complete clinicopathological variables required for ML model development, resulting in a final study population of 459 patients. For predictive modeling, clinical outcomes were recoded into a binary recurrence status variable, and patients were categorized as recurrent or nonrecurrent. Although the original METABRIC endpoint represents PFS as a time-to-event outcome, the present machine learning analysis was designed as a binary classification task. Therefore, PFS information was operationalized as a binary recurrence status (recurrent versus nonrecurrent), irrespective of the exact time to progression. Accordingly, the developed machine learning models should be interpreted as recurrence classifiers rather than time-to-event survival prediction models. Principal component analysis (PCA) was performed to assess overall transcriptomic variation and evaluate potential technical confounding. Sample clustering was primarily associated with biological subgroup characteristics, particularly age-defined subgroup classification and recurrence status. No substantial batch-related confounding was observed in subsequent analyses.

### 2.3. Differentially Expressed Gene (DEG) Analysis

The analytical workflow used in the present study was based on established transcriptomic data mining strategies previously applied in cancer-related gene expression studies [38]. To investigate age-related differences, Luminal A BC patients in the METABRIC cohort were divided into three groups: premenopausal, postmenopausal non-geriatric, and geriatric (≥70 years). Differential gene expression analyses were performed using linear modeling implemented in the limma package in R (version 4.2.3). DEG comparisons were conducted at two analytical levels. First, within each age-defined subgroup, patients with documented recurrence were compared with nonrecurrent patients to identify recurrence-associated transcriptomic alterations within each age-defined subgroup. Second, recurrence-positive and recurrence-negative patients were compared across the entire study cohort to identify global recurrence-associated expression patterns. Genes were considered differentially expressed if they met the thresholds of Benjamini–Hochberg (BH) adjusted false discovery rate (FDR) ≤ 0.1 and absolute log_2_ fold change (|log_2_FC|) ≥ 1.

To evaluate the robustness of the identified differentially expressed genes, a sensitivity analysis was additionally performed using a more stringent BH-adjusted FDR threshold of ≤0.05. The principal candidate genes remained statistically significant under this stricter criterion, and the corresponding results are presented in Table S1.

### 2.4. KEGG Pathway Enrichment Analysis

To further explore the biological pathways associated with recurrence-related transcriptomic alterations, Kyoto Encyclopedia of Genes and Genomes (KEGG) pathway enrichment analysis was performed separately for upregulated and downregulated DEGs using the clusterProfiler package in R (version 4.2.3). Statistically significant pathways were identified based on BH false discovery rate (FDR)-adjusted *p*-values. The enriched pathways were visualized according to enrichment significance (−log_10_ FDR), gene count, and fold enrichment.

### 2.5. External Validation Analysis

To evaluate the robustness of the principal candidate genes identified in the METABRIC discovery cohort, an independent external validation analysis was performed using the Gene Expression Omnibus (GEO) dataset GSE20685. Gene expression data were generated using the GPL570 (Affymetrix Human Genome U133 Plus 2.0 Array, Affymetrix, Santa Clara, CA, USA) platform. Based on the published molecular subtype mapping of GSE20685, subtype V and subtype VI samples were considered Luminal A-like, resulting in a validation cohort of 134 patients. Because GSE20685 does not provide the same composite PFS endpoint or the complete clinicopathological information available in the METABRIC cohort, validation was performed at the gene level rather than at the machine learning model level. Accordingly, the prognostic relevance of the principal candidate genes identified in the discovery phase was evaluated in the independent cohort. In the GSE20685 validation cohort, metastasis-free survival was defined using the *event_metastasis* variable together with the corresponding follow-up duration provided in the original dataset. Associations between gene expression and metastasis-free survival were evaluated using Cox proportional hazards regression analysis, and hazard ratios (HRs), 95% confidence intervals (95% CIs), and corresponding *p*-values were calculated.

### 2.6. Principal Component Analysis

PCA was performed to assess the overall variation in gene expression profiles and to explore whether transcriptomic patterns differed according to age-defined subgroups and recurrence status. The analysis was conducted on centered and scaled gene expression values using the prcomp function in R software (version 4.2.3), and visualizations were generated using the ggplot2 package. In the PCA plots, age-defined subgroups were indicated by different colors, whereas recurrence status was represented using distinct symbols to facilitate subgroup comparison.

### 2.7. Statistical Analysis

Statistical analyses were performed on the final cohort of 459 patients with complete clinical and clinicopathological annotations. Patients were compared according to recurrence status, defined as nonrecurrent and recurrent groups. Continuous variables are presented as mean ± standard deviation, whereas categorical variables are presented as frequencies and percentages. The normality of continuous variables was assessed using the Kolmogorov–Smirnov test, and homogeneity of variances was evaluated using Levene’s test. For normally distributed continuous variables with homogeneous variances, comparisons between recurrent and nonrecurrent groups were performed using the independent samples *t*-test. Categorical variables were compared using the chi-square test. A *p*-value of <0.05 was considered statistically significant. All statistical analyses were performed using Jamovi software (version 2.4.6).

### 2.8. Machine Learning

ML comprises a set of computational approaches capable of learning complex relationships from large-scale datasets and generating predictive models for clinical outcome assessment [38,39]. Owing to their ability to integrate high-dimensional molecular and clinical information, ML methods have become increasingly valuable in cancer prognostic research [40,41]. In this study, the following analytical steps were implemented to develop ML models for classifying binary recurrence status (recurrent vs. nonrecurrent), which was derived from the original PFS endpoint.

### 2.9. Prediction Models

In this study, Logistic Regression (LR), Multilayer Perceptron (MLP), Random Forest (RF), and XGBoost algorithms were used to classify recurrence status (recurrent vs. nonrecurrent) in Luminal A BC patients. LR is widely used in clinical research to model binary outcomes and estimate the independent contribution of predictors using odds ratios [39]. MLP is an artificial neural network model capable of learning nonlinear relationships through input, hidden, and output layers [40]. RF improves prediction performance by generating multiple decision trees using the random sampling (bagging) method and limits overfitting [41]. XGBoost is an efficient implementation of gradient-augmented decision trees that prevents overfitting through adjustment techniques and is widely applied in biomedical data analysis [42].

The final dataset consisted of 267 nonrecurrent and 192 recurrent patients. The dataset was randomly divided into training and independent testing subsets using a 70:30 split strategy. To address the potential impact of class imbalance, the Synthetic Minority Oversampling Technique (SMOTE) was initially evaluated within the training set during 5-fold cross-validation. SMOTE generates synthetic minority-class samples based on feature-space similarity and was assessed as a potential strategy to improve model performance [43]. However, because the class distribution was only mildly imbalanced and no meaningful improvement in predictive performance was observed, all final models were developed using the original, non-oversampled dataset. The independent test set was never subjected to oversampling and retained its original class distribution throughout model evaluation.

Feature selection was performed using the Boruta algorithm based on Gini RF importance, and this procedure was applied only to the training set. To evaluate the incremental predictive value of transcriptomic biomarkers, an additional baseline analysis was performed using only conventional clinicopathological variables (age at diagnosis, tumor size, NPI, type of breast surgery, radiotherapy status, histological grade, histopathological subtype, and tumor stage). These baseline models were constructed using the same machine learning workflow as the integrated models, including identical training/testing partitions, stratified 5-fold cross-validation, hyperparameter optimization, and performance evaluation metrics. Their performance is presented in Table S2. To evaluate the stability of the Boruta feature selection procedure, an additional bootstrap stability analysis was performed. The Boruta algorithm was repeated across 100 bootstrap resamples, and the selection frequency, mean Random Forest importance, and standard deviation of feature importance were calculated for each variable. Based on the bootstrap selection frequency, features were classified as having high (≥80%), moderate (50–79%), or low (<50%) stability. All modeling and analysis steps were performed using R software (version 4.2.3). LR was implemented using the glm() function, while RF, MLP, and XGBoost models were implemented using the RF, nnet, and XGBoost packages, respectively. Model performance was evaluated using accuracy, sensitivity, specificity, F1-score, and area under the receiver operating characteristic curve (AUC). Hyperparameter optimization was performed using grid search within the caret package.

## 3. Result

### 3.1. Global Gene Expression Variation and Subgroup Clustering Identified by PCA

**Figure 2:**
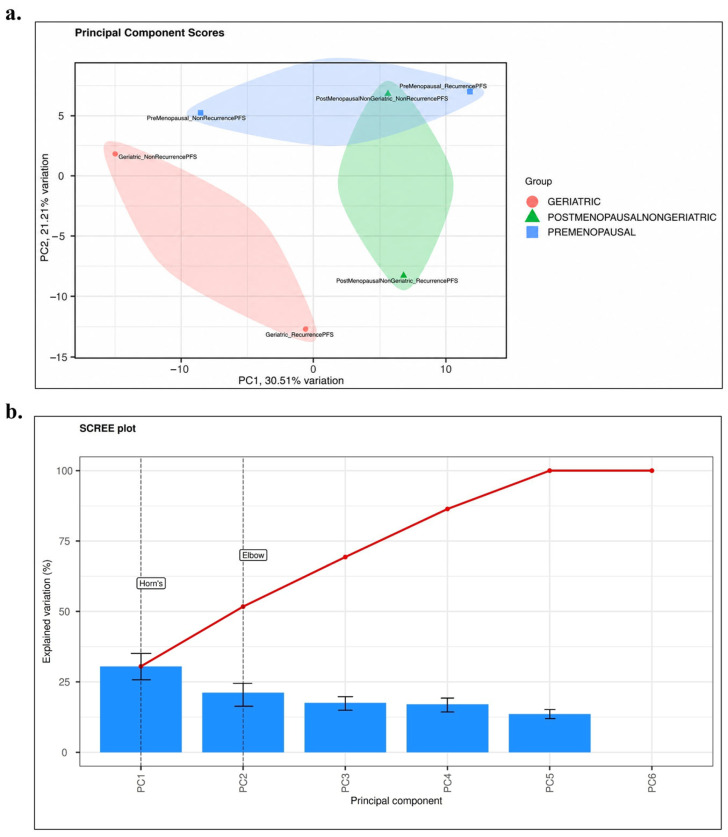
PCA results by PFS status and age groups. (**a**) The PCA score plot showed partial separation of patient subgroups by age group and recurrence status. The points represent individual samples, with colors indicating age groups and symbols indicating recurrence status. The ellipses represent the 95% CIs for each group. The first two principal components, PC1 (30.51%) and PC2 (21.2%), explain a significant portion of the total variance and show a distinct separation between subgroups. (**b**) The scree plot analysis shows the percentage of variance explained by each principal component. According to the elbow criterion and Horn’s parallel analysis, PC1 and PC2 were identified as the most meaningful components, and it was observed that the contribution of subsequent components decreases gradually.

PCA was performed to examine the overall variation in gene expression profiles and to evaluate clustering patterns in patient subgroups classified by age and recurrence status. The first two principal components, PC1 and PC2, explained 30.51% and 21.2% of the total variance, respectively, and together accounted for 51.71% of the total variability in the dataset (Figure 2a). The PCA score plot demonstrated that patient subgroups were partially separated based on both age classification and recurrence status. The premenopausal, postmenopausal–non-geriatric, and geriatric groups formed distinct clusters, suggesting that age-related transcriptomic differences contribute to global gene expression patterns. Within each age group, the subclassification according to recurrence status contributed to the intra-group distribution, suggesting the presence of recurrence-associated transcriptional differences.

The geriatric group showed a wider spread along the negative PC2 axis, especially among recurrent cases, indicating increased transcriptomic variability in this subgroup. In contrast, premenopausal patients exhibited tighter clustering, particularly in the subgroup without recurrence, indicating a relatively more homogeneous gene expression profile. The postmenopausal–non-geriatric group, however, showed intermediate variability, with a shift along the PC2 axis observed in samples associated with recurrence. A scree plot analysis was performed to evaluate the contribution of the principal components in greater detail (Figure 2b). The results confirmed that PC1 and PC2 were the most informative components, as validated by both the elbow criterion and the Horn parallel analysis test. The contribution of other components to variance decreased gradually, supporting the appropriateness of using the first two components in subsequent analyses. These findings suggest that transcriptomic variation in Luminal A BC is associated with both age-defined subgroup and recurrence status. These clustering patterns provided support for subsequent differential expression and ML analyses.

To evaluate the potential influence of technical variation on transcriptomic profiles, batch effect assessment was performed using PCA following log_2_ transformation and normalization of the gene expression data. PCA conducted on centered and scaled expression values indicated that sample clustering was primarily associated with biological subgroup characteristics, particularly age group and recurrence status. Given that the METABRIC cohort was generated using a single microarray platform (Illumina HumanHT-12 v3) and standardized processing procedures, no substantial batch-related effects were detected. Therefore, no additional batch correction was applied in subsequent analyses.

### 3.2. Recurrence-Associated Gene Expression Patterns Across Age-Defined Luminal A Patient Groups

**Figure 3:**
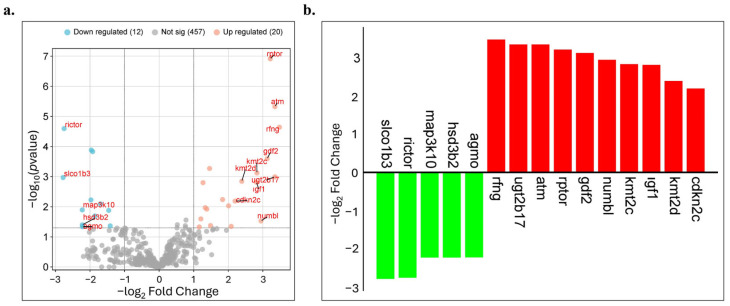
Recurrence-associated differential gene expression patterns across age-defined Luminal A BC patient groups. (**a**) Volcano plot illustrating DEGs between comparison groups. The x-axis represents log_2_ fold change, and the y-axis represents −log_10_(*p*-value). Upregulated genes are shown in red, downregulated genes in blue, and non-significant genes in gray. (**b**) Bar plot showing log_2_ fold change values of selected significantly dysregulated genes. Upregulated genes are presented in red, while downregulated genes are shown in green, demonstrating distinct expression trends across the analyzed groups.

Differential gene expression analysis was performed to identify recurrence-associated transcriptomic alterations across age-defined Luminal A patient groups. To provide a comprehensive overview of transcriptomic alterations at both global and gene-specific levels, volcano plot visualization and fold-change analyses of selected genes were performed (Figure 3). In addition, statistical significance and expression direction of the selected genes are summarized in Table 2.

Differential expression analysis initially identified 32 significantly dysregulated genes (20 upregulated and 12 downregulated) using thresholds of FDR ≤ 0.1 and |log_2_FC| ≥ 1, whereas the majority of transcripts remained non-significant. As illustrated in the volcano plot (Figure 3a), the majority of transcripts exhibited limited expression changes and did not reach statistical significance, whereas a distinct subset of genes showed marked upregulation or downregulation, indicating recurrence-associated transcriptomic alterations.

To focus on the most prominent transcriptomic alterations and facilitate data visualization, a more stringent threshold of |log_2_FC| ≥ 2 was subsequently applied. This additional filtering step reduced the candidate gene set to 15 genes, comprising 10 upregulated and 5 downregulated transcripts, which were selected for detailed presentation in Figure 3b and Table 2. A sensitivity analysis using a more stringent BH-adjusted FDR threshold of ≤0.05 confirmed that the principal candidate genes, including Radical Fringe O-fucosylpeptide 3-beta-N-acetylglucosaminyltransferase *(RFNG)*, Ataxia-Telangiectasia Mutated *(ATM)*, and lysine methyltransferase 2D *(KMT2D)*, remained statistically significant (Table S1), indicating that the main biological conclusions were robust to the choice of FDR threshold.

Among the significantly upregulated genes, *RFNG* exhibited the highest expression increase with a log_2_ fold change value of 3.4298 (FDR < 0.001), followed by UDP glucuronosyltransferase family 2 member B17 (*UGT2B17*) (log_2_FC = 3.3504, FDR < 0.001), *ATM* (log_2_FC = 3.3491, FDR < 0.001), Regulatory Associated Protein Of MTOR Complex 1 (*RPTOR*) (log_2_FC = 3.2156, FDR < 0.001), and Growth Differentiation Factor 2 (*GDF2*) (log_2_FC = 3.1251, FDR < 0.001) (Table 2). Additional upregulated genes included NUMB-like endocytic adaptor protein (*NUMBL)* (log_2_FC = 2.9478, FDR < 0.001), Lysine Methyltransferase 2C (*KMT2C*) (log_2_FC = 2.8304, FDR < 0.001), Insulin-like growth factor 1 (*IGF1*) (log_2_FC = 2.8135, FDR < 0.001), *KMT2D* (log_2_FC = 2.3912, FDR = 0.014), and Cyclin Dependent Kinase Inhibitor 2C (*CDKN2C*)(log_2_FC = 2.1967, FDR = 0.026).

Conversely, several genes showed significant downregulation across the analyzed groups (Figure 3b; Table 2). Solute carrier organic anion transporter family member 1B3 (*SLCO1B3*) demonstrated the strongest decrease in expression (log_2_FC = −2.7775, FDR < 0.001), followed by RPTOR-independent companion of MTOR complex (*RICTOR*) (log_2_FC = −2.7493, FDR < 0.001), Mitogen-activated protein kinase kinase kinase 10 (*MAP3K10*) (log_2_FC = −2.2253, FDR = 0.018), Hydroxy-Delta-5-Steroid Dehydrogenase, 3 Beta- And Steroid Delta-Isomerase 2 (*HSD3B2*)(log_2_FC = −2.2235, FDR = 0.019), and Alkylglycerol monooxygenase (*AGMO*) (log_2_FC = −2.2195, FDR = 0.021).

Figure 3b further illustrates the magnitude and direction of expression changes observed in the selected genes, highlighting distinct recurrence-associated transcriptional differences between the upregulated and downregulated gene sets. Collectively, these findings demonstrate the presence of recurrence-associated transcriptomic alterations across age-defined Luminal A patient groups. The expression profiles summarized in Figure 3 and Table 2 were subsequently incorporated into downstream prognostic and machine learning analyses.

**Figure 4:**
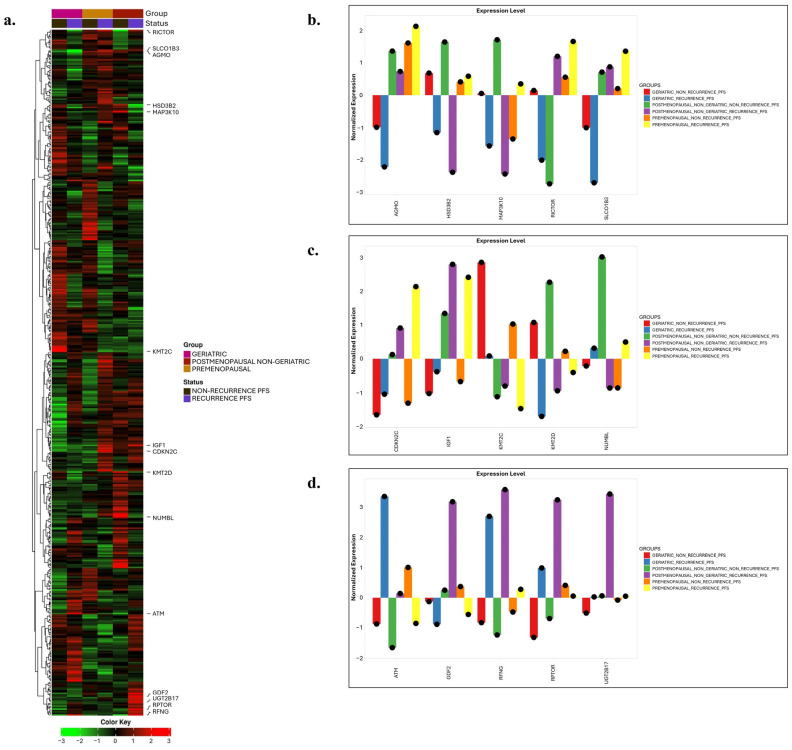
Multidimensional visualization of differential gene expression patterns associated with PFS in patients with Luminal A BC. (**a**) A heatmap generated via hierarchical clustering of genes found to be statistically significant across all samples. Rows represent genes, and columns represent patient samples. The color scale indicates normalized gene expression levels, with red representing high expression and green representing low expression. The annotations in the top panel indicate the age groups of the samples (premenopausal, postmenopausal–non-geriatric, geriatric) and their recurrence status (recurrence/no recurrence). (**b**–**d**) Bar charts showing the normalized expression levels of selected genes according to age groups and recurrence status. Each panel represents a different set of genes, revealing that genes exhibit distinct expression patterns across subgroups. These charts provide a detailed view of how specific genes change in relation to progression and age.

The direction of change in expression reflects the overall trend identified in the global recurrence-associated differential expression analysis, whereas age- and subgroup-specific expression patterns are presented separately in Figure 4.

### 3.3. Identification of Genes Differentially Expressed in Relation to PFS

Differential gene expression analysis identified a distinct set of recurrence-associated genes, revealing substantial transcriptomic differences between patients with and without disease progression. Both upregulated and downregulated transcripts were detected, indicating that PFS is associated with coordinated molecular alterations in Luminal A BC. Subsequent KEGG pathway enrichment analysis was performed to explore the biological pathways associated with these transcriptomic alterations.

Age-stratified analyses demonstrated that the transcriptomic impact of disease progression was not uniform across patient groups. Differential expression patterns were relatively limited in premenopausal patients, consistent with the tighter clustering observed in the PCA. In contrast, more pronounced transcriptional differences were detected between recurrent and nonrecurrent cases in the geriatric group, suggesting greater molecular heterogeneity associated with disease progression in older patients. The postmenopausal non-geriatric group exhibited an intermediate profile, with distinguishable but less extensive expression differences compared with the geriatric cohort.

These observations were supported by the heatmap and subgroup-specific expression analyses presented in Figure 4. Several genes exhibited distinct age-dependent expression patterns, whereas others showed consistent recurrence-associated trends across all patient groups. The concordance between PCA clustering patterns, differential expression results, and subgroup-specific gene expression profiles supports the biological relevance of the identified transcriptomic signatures.

These findings suggest that recurrence-associated transcriptomic alterations in Luminal A BC exhibit age-dependent expression patterns. The identified candidate genes were subsequently subjected to KEGG pathway enrichment analysis, independent external validation, and machine learning-based predictive modeling.

A heatmap analysis based on hierarchical clustering was performed to evaluate the distribution of DEGs across patient subgroups (Figure 4a). Distinct expression patterns were observed among the age-defined and recurrence-based groups, indicating that both age and disease progression contribute to transcriptomic heterogeneity in Luminal A BC. Although subgroup separation was not absolute, several clusters displayed characteristic expression profiles associated with specific patient categories.

An analysis of the genes shown in Figure 4b (*AGMO*, *HSD3B2*, *MAP3K10*, *RICTOR*, and *SLCO1B3*) reveals that their expression patterns differ significantly across age groups and recurrence status. In the *AGMO* gene, expression increased upon transition from the geriatric group to the postmenopausal–non-geriatric group; additionally, a distinct upward trend emerged in the premenopausal group in conjunction with recurrence. In the *HSD3B2* gene, a marked decrease is observed in the transition from non-recurrent to recurrent cases, particularly in the postmenopausal–non-geriatric group, while in the geriatric group, expression is generally low and is further suppressed in the presence of recurrence. In the *MAP3K10* gene, lower expression was observed in the postmenopausal–non-geriatric group in cases of recurrence, while a slight upward trend was noted in the premenopausal group in cases of recurrence. In the *RICTOR* gene, different directional changes were observed across age groups: while higher expression was observed in the postmenopausal–non-geriatric group in the presence of recurrence, expression was suppressed in the geriatric group in the presence of recurrence. In the *SLCO1B3* gene, a suppressed profile is maintained in the geriatric group, while an increasing trend in conjunction with recurrence is notable in the postmenopausal–non-geriatric and premenopausal groups.

Analysis of the genes in Figure 4c (*CDKN2C*, *IGF1*, *KMT2C*, *KMT2D*, and *NUMBL*) reveals that their expression patterns show clearer group-specific differences. In the *CDKN2C* gene, a shift from negative to positive expression in conjunction with recurrence is particularly notable in the premenopausal group. In the *IGF1* gene, an upward trend in expression was observed across all age groups in conjunction with recurrence, though this increase was more pronounced in the premenopausal group. In the *KMT2C* gene, expression decreased in both the geriatric and premenopausal groups in conjunction with recurrence, while a more limited change was observed in the postmenopausal–non-geriatric group. In the *KMT2D* gene, expression levels decreased with recurrence in all three age groups, and a shift from positive to negative was observed in some groups. In the *NUMBL* gene, the direction of expression change shifts with age; while a lower expression was observed in the postmenopausal–non-geriatric group in conjunction with recurrence, a trend toward a shift from negative to positive was observed in the geriatric and premenopausal groups. The genes shown in Figure 4d (*ATM*, *GDF2*, *RFNG*, *RPTOR*, and *UGT2B17*) also display notable changes that vary with age and recurrence status. A change in direction associated with recurrence is observed in the *ATM* gene across all age groups. In the *GDF2* gene, higher expression is observed with recurrence in the postmenopausal–non-geriatric group, whereas the opposite trend is seen in the premenopausal group. In the *RFNG* gene, a distinct pattern of higher expression with recurrence emerges across all age groups. In the *RPTOR* gene, an increase is observed alongside recurrence in the geriatric and postmenopausal–non-geriatric groups, while a more limited change is seen in the premenopausal group. In the *UGT2B17* gene, the most pronounced change is observed in the postmenopausal–non-geriatric recurrence group.

These findings reveal age-dependent recurrence-associated gene expression patterns, with *KMT2D*, *RFNG*, *IGF1*, *CDKN2C*, *ATM*, and *RPTOR* emerging as important variables in subsequent ML analyses.

### 3.4. Biological Pathways Associated with Recurrence-Related Transcriptomic Alterations

**Figure 5:**
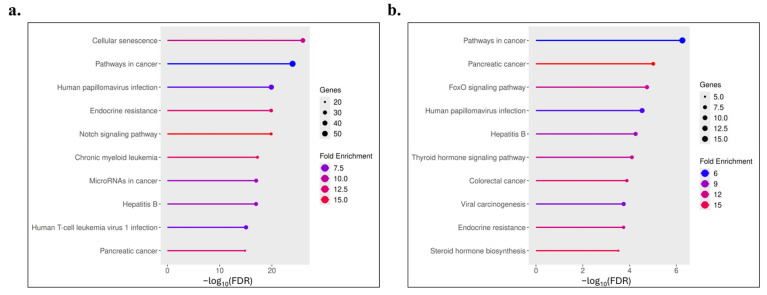
KEGG pathway enrichment analysis of recurrence-associated differentially expressed genes (DEGs) in Luminal A breast cancer. (**a**) Top enriched KEGG pathways identified among upregulated DEGs. (**b**) Top enriched KEGG pathways identified among downregulated DEGs. Bubble size represents the number of DEGs mapped to each pathway, whereas color represents fold enrichment (blue = lower; red = higher). The x-axis represents pathway significance expressed as −log_10_(FDR).

To further investigate the biological pathways associated with recurrence-related transcriptomic alterations, KEGG pathway enrichment analysis was performed separately for upregulated and downregulated differentially expressed genes (DEGs) (Figure 5; Table 3). Among the upregulated DEGs, significant enrichment was observed in pathways related to cellular senescence, pathways in cancer, human papillomavirus infection, endocrine resistance, Notch signaling, microRNAs in cancer, hepatitis B, and pancreatic cancer. In contrast, downregulated DEGs were primarily enriched in pathways in cancer, FoxO signaling, thyroid hormone signaling, endocrine resistance, and viral carcinogenesis. Overall, these findings provide biological context for the recurrence-associated transcriptomic alterations identified in Luminal A BC and suggest that these alterations are associated with multiple biological pathways related to tumor progression, cellular senescence, growth factor signaling, endocrine regulation, and cell fate determination.

KEGG pathway enrichment analysis was performed separately for upregulated and downregulated DEGs. Pathways are ranked according to FDR-adjusted significance. Gene count indicates the number of DEGs mapped to each pathway.

### 3.5. External Validation of the Principal Candidate Genes

To further evaluate the robustness of the principal candidate genes identified in the METABRIC discovery cohort, an independent external validation analysis was performed using the GEO dataset GSE20685. Based on the published molecular subtype classification, subtype V and subtype VI samples were considered Luminal A-like and included in the validation cohort (Table 4). Because GSE20685 does not provide the same composite PFS endpoint or the complete clinicopathological information available in the METABRIC cohort, validation was performed at the gene level using Cox proportional hazards regression analysis for metastasis-free survival. As summarized in Table 5, *KMT2D* (HR = 0.421, 95% CI: 0.179–0.992, *p* = 0.048), *RFNG* (HR = 0.481, 95% CI: 0.206–0.524, *p* = 0.038), *ATM* (HR = 0.341, 95% CI: 0.099–0.586, *p* = 0.018), and *RICTOR* (HR = 1.016, 95% CI: 1.001–1.032, *p* = 0.042) remained significantly associated with clinical outcome in the independent validation cohort. In contrast, *IGF1*, *CDKN2C*, and *RPTOR* were not significantly associated with metastasis-free survival (*p* > 0.05). Despite differences in clinical endpoints between the discovery and validation cohorts, these findings provide independent support for the robustness of several principal transcriptomic biomarkers identified in the METABRIC discovery cohort, although further validation in larger independent patient cohorts using comparable clinical endpoints is warranted.

Subtype V and subtype VI were considered Luminal A-like according to the published molecular subtype classification and were included in the external validation analysis.

### 3.6. Statistical Analysis of Survival Status Across Geriatric, Premenopausal, and Postmenopausal Patients

As shown in Table 6, recurrent patients were older than nonrecurrent patients, with mean ages of 61.1 and 57.4 years, respectively (*p* < 0.001; 95% CI: 0.12–0.50). Similarly, tumor size was larger in the recurrent group (*p* < 0.001; 95% CI: 0.23–0.60). Follow-up duration was shorter among recurrent patients, with mean values of 85.5 and 151.1 months, respectively (*p* < 0.001; 95% CI: −1.15 to −0.76). In addition, NPI values were higher in recurrent patients (*p* < 0.001; 95% CI: 0.27–0.65).

According to the results presented in Table 7, recurrence status was significantly associated with breast surgery type (*p* < 0.001; Cramer’s V = 0.157), radiotherapy status (*p* = 0.020; Cramer’s V = 0.108), histological grade (*p* < 0.001; Cramer’s V = 0.174), histopathological subtype (*p* = 0.024; Cramer’s V = 0.093), and tumor stage (*p* = 0.002; Cramer’s V = 0.176). No significant associations were found for chemotherapy, primary tumor laterality, cellularity, or HER2 loss snp 6 status (*p* > 0.05). Overall, these findings indicate that several clinicopathological variables, including surgical approach, radiotherapy status, histological grade, histopathological subtype, and tumor stage, are significantly associated with recurrence status in Luminal A BC patients.

### 3.7. Machine Learning-Based and Ensemble Classification of Breast Cancer Survival Outcomes

To investigate the predictive potential of integrated clinicopathological and transcriptomic variables for recurrence classification derived from the PFS endpoint in Luminal A BC patients, multiple supervised ML algorithms were developed and comparatively evaluated. The dataset was randomly divided into training and testing cohorts using a 70:30 split strategy. Stratified 5-fold cross-validation was employed during model training to improve model stability and reduce the risk of overfitting.

**Figure 6:**
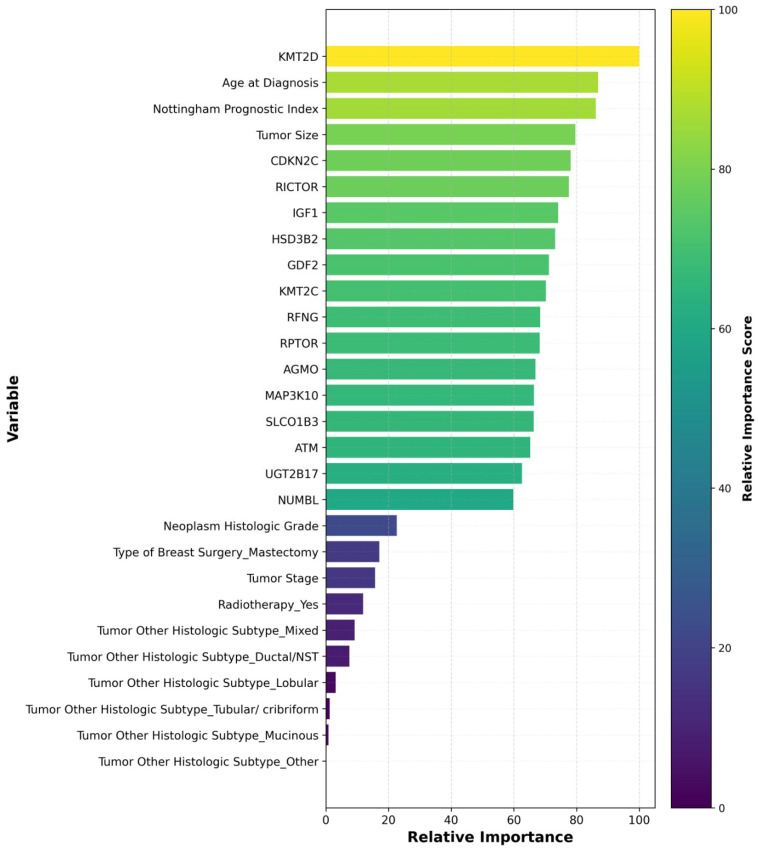
Relative importance of clinical variables and DEGs identified using the Boruta feature selection algorithm for recurrence risk classification in Luminal A BC patients. Variables are ranked according to their relative importance scores. *KMT2D* demonstrated the highest predictive contribution, followed by key clinical parameters including age at diagnosis, NPI, and tumor size, together with several transcriptomic biomarkers.

Feature selection using the Boruta algorithm identified a subset of highly informative clinical and molecular predictors associated with recurrence status classification (Figure 6). Among all evaluated variables, *KMT2D* demonstrated the highest relative importance score, indicating a potentially dominant role in model discrimination. Among clinical parameters, age at diagnosis, NPI, and tumor size emerged as the most influential predictors. Several transcriptomic biomarkers also showed substantial predictive importance, including *CDKN2C*, *RICTOR*, *IGF1*, *HSD3B2*, *GDF2*, *KMT2C*, *RFNG*, *RPTOR*, *AGMO*, *MAP3K10*, *SLCO1B3*, *ATM*, *UGT2B17*, and *NUMBL*. Compared with the top-ranked predictors, histological subtype-related variables, radiotherapy status, tumor stage, and breast surgery type showed lower relative importance scores. These findings suggest that recurrence status prediction in Luminal A BC is strongly influenced by both conventional clinical indicators and recurrence-associated transcriptomic signatures. To further evaluate the robustness of the Boruta feature selection procedure, a bootstrap stability analysis was performed using 100 bootstrap resamples (Table S3). The principal predictors remained consistently selected across bootstrap iterations. NPI (98%), age at diagnosis (92%), tumor size (85%), and *KMT2D* (80%) demonstrated high selection stability, whereas the remaining transcriptomic biomarkers generally exhibited moderate selection stability (50–79%). These findings support the robustness and reproducibility of the identified feature set. Hyperparameter optimization was performed for all ML models using grid-search-based tuning strategies, and the optimized parameter combinations are summarized in Table 8.

**Figure 7:**
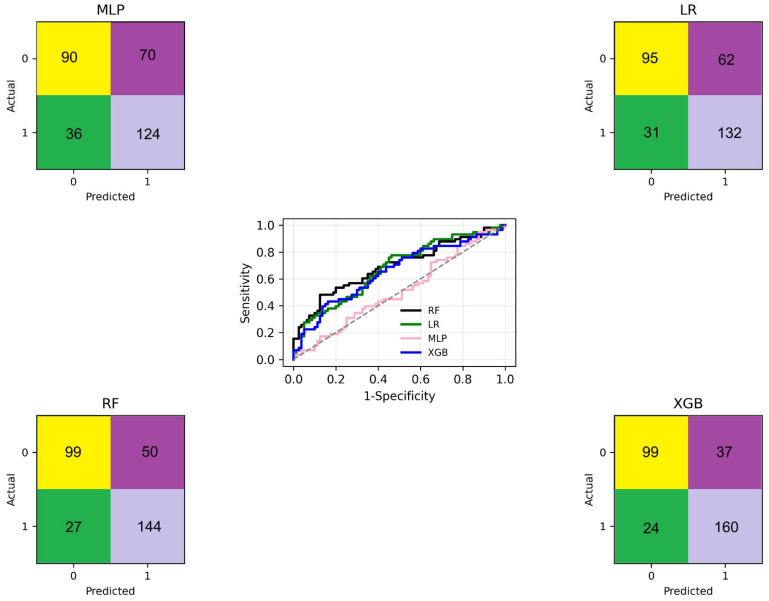
Comparative training-phase performance of ML classifiers for recurrence classification in Luminal A BC patients. Confusion matrices for MLP, LR, RF, and XGBoost (XGB) models are shown together with ROC curve analysis. Ensemble-based classifiers, particularly XGBoost and RF, demonstrated superior discrimination performance during training.

During training-phase evaluation, differences in model discrimination performance were observed (Figure 7). Among all classifiers, XGBoost (XGB) achieved the best overall performance, with the highest diagnostic accuracy (0.80), sensitivity (0.81), F1-score (0.84), positive predictive value (0.87), and AUC = 0.72. The XGB confusion matrix showed 160 true positive predictions with 24 false negatives, indicating good sensitivity for identifying progression-associated cases. Similarly, the RF model demonstrated good predictive performance, with a diagnostic accuracy of 0.76, sensitivity of 0.74, specificity of 0.79, and F1-score of 0.79, correctly identifying 144 true positive cases while maintaining a relatively balanced false classification profile.

The LR model showed intermediate performance, with diagnostic accuracy of 0.71, sensitivity of 0.68, specificity of 0.75, and AUC of 0.68, whereas the MLP demonstrated comparatively weaker performance, yielding the lowest AUC (0.58) and overall classification accuracy (0.69). Receiver Operating Characteristic (ROC) analysis during training supported these observations, with ensemble-based methods consistently outperforming conventional classifiers.

**Figure 8:**
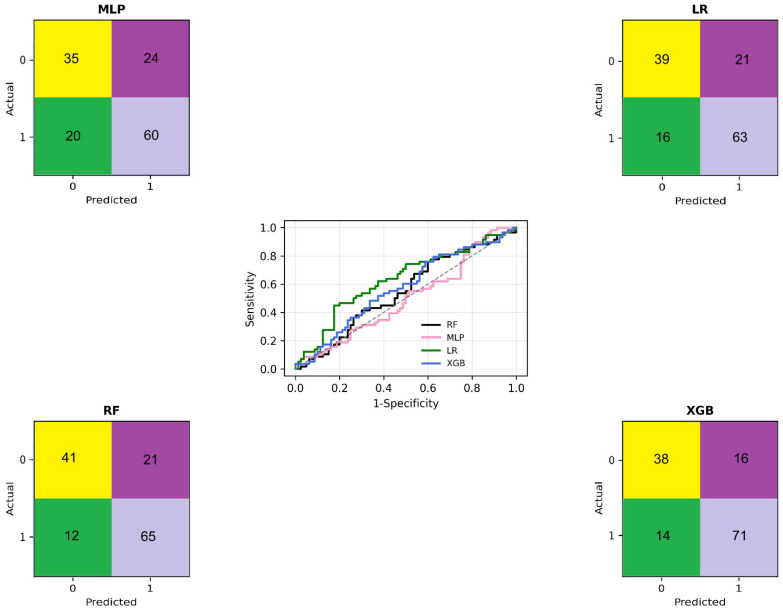
Comparative testing-phase performance of ML classifiers for recurrence classification in Luminal A BC patients. Confusion matrices and ROC curve analysis for independent testing data are shown for MLP, LR, RF, and XGBoost (XGB) models. XGBoost and RF maintained the strongest predictive performance, indicating good generalizability and stable classification capability.

Independent testing on previously unseen data supported the generalizability of the developed models (Figure 8; Table 9). Among all evaluated classifiers, XGBoost maintained the strongest predictive performance, achieving the highest sensitivity (0.82), F1-score (0.83), diagnostic accuracy (0.78), and negative predictive value (0.71), together with a stable AUC (0.71). The corresponding confusion matrix demonstrated 71 true positive predictions, 38 true negatives, 16 false positives, and 14 false negatives, indicating sustained discrimination capacity in the independent test set.

The RF model also demonstrated strong generalizability, with diagnostic accuracy of 0.76, specificity of 0.77, F1-score of 0.80, and the highest positive predictive value (0.84), correctly classifying 65 true positive cases with only 12 false negatives, reflecting the lowest false negative burden among all evaluated models. The LR model maintained moderate performance, achieving an AUC of 0.71 with balanced sensitivity (0.75) and specificity (0.71), whereas the MLP model remained the weakest classifier, with lower discrimination metrics across nearly all performance measures.

To further evaluate the incremental predictive value of transcriptomic biomarkers, additional baseline machine learning models were developed using only conventional clinicopathological variables (Table S3). Among these models, logistic regression achieved the highest discrimination in the independent testing cohort (AUC = 0.674, 95% CI: 0.574–0.756). By comparison, the integrated clinicopathological–transcriptomic XGBoost model achieved a higher AUC (0.71), together with improved diagnostic accuracy, sensitivity, and F1-score. These findings indicate that the incorporation of transcriptomic biomarkers provided additional predictive information beyond conventional clinicopathological variables, although the magnitude of improvement was moderate.

The relatively consistent performance observed between the training and independent testing cohorts suggests that the developed models achieved satisfactory generalizability while minimizing the risk of substantial overfitting. Taken together, these results highlight the utility of integrating clinicopathological variables with transcriptomic biomarkers for ML-based classification of progression-related outcomes in Luminal A BC, with ensemble learning approaches, most notably XGBoost and RF, emerging as the most reliable predictive models.

## 4. Discussion

In the present study, transcriptomic alterations associated with PFS in Luminal A BC were investigated using an age-stratified approach. PCA suggested that age was a major contributor to global gene expression variability, while recurrence status contributed additional molecular separation within each age group. The broader transcriptomic dispersion observed among geriatric patients suggests greater molecular heterogeneity associated with recurrence, whereas premenopausal patients exhibited comparatively more homogeneous transcriptomic profiles. These findings suggest that age is not merely a clinical variable in Luminal A BC but also an important transcriptomic determinant shaping tumor biology. Among the DEGs identified in this study, *KMT2D*, *RFNG*, *IGF1*, and *CDKN2C* exhibited the most consistent recurrence-associated expression patterns across all three age groups, whereas *ATM*, *RPTOR*, and *RICTOR* displayed subgroup-dependent expression profiles, suggesting that their association with recurrence may vary across age-defined patient subgroups. Together, these findings indicate that the molecular mechanisms underlying recurrence in Luminal A BC may include both shared and subgroup-dependent components.

KMT2D is a major histone H3 lysine 4 (H3K4) monomethyltransferase that plays a critical role in enhancer activation and the regulation of chromatin architecture [44,45]. Previous studies have suggested that KMT2D functions as a tumor suppressor by maintaining epigenetic homeostasis and regulating p53-mediated DNA damage responses, thereby contributing to genomic integrity and tumor suppression. [46]. In addition, *KMT2D* mutations have also been identified in BC brain metastases, supporting its potential involvement in tumor progression and metastasis [47]. In our study, *KMT2D* expression was consistently reduced in patients who experienced recurrence across all age-defined patient subgroups and was identified as the most important feature in the Boruta analysis. The consistent recurrence-associated expression pattern of *KMT2D*, together with its high predictive importance, suggests that it represents one of the most promising biomarker candidates identified in this study. These findings support the notion that epigenetic regulatory mechanisms may contribute to recurrence development in Luminal A BC. Consistent with these observations, KEGG pathway enrichment analysis demonstrated significant enrichment of cellular senescence and pathways in cancer, providing additional biological context for the identified transcriptomic alterations. The Notch signaling pathway regulates cell proliferation, differentiation, and tissue homeostasis, and its dysregulation has been implicated in the development and progression of numerous malignancies [48,49]. RFNG is a glycosyltransferase that modulates Notch receptor signaling and contributes to the regulation of Notch pathway activity [50,51]. In addition, elevated *RFNG* expression has been reported in several tumor types and may be associated with tumor progression [52]. In our study, *RFNG* was consistently upregulated in patients who experienced recurrence across age-defined patient subgroups. This consistent expression pattern suggests that *RFNG*-associated Notch signaling may contribute to recurrence irrespective of patient subgroup. Consistent with these findings, KEGG pathway enrichment analysis also identified significant enrichment of the Notch signaling pathway among the upregulated differentially expressed genes. IGF-1 is a key mitogenic growth factor that primarily signals through the IGF-1 receptor, promoting cell proliferation, epithelial-to-mesenchymal transition, and inhibition of apoptosis. Increasing evidence implicates IGF-1 signaling in breast cancer development, progression, and metastasis [53]. In the present study, *IGF1* expression was consistently elevated in patients who experienced recurrence across all age-defined patient subgroups, suggesting that growth factor-mediated signaling may contribute to disease progression and PFS-related events in Luminal A BC. Consistent with these findings, KEGG pathway enrichment analysis identified significant enrichment of endocrine resistance and cancer-related pathways, further supporting the involvement of growth factor-mediated signaling in recurrence-associated transcriptomic alterations. The similar expression patterns observed for *RFNG* and *IGF1* across age-defined patient subgroups suggest that these genes may participate in shared recurrence-associated biological processes.

CDKN2C is a member of the INK4 family of cyclin-dependent kinase inhibitors and contributes to G1 cell cycle arrest through the inhibition of CDK4 and CDK6 activity [54,55,56]. Previous studies have associated CDKN2C with tumor biology and clinical prognosis in several malignancies [55,57,58]. In our study, *CDKN2C* exhibited a consistent recurrence-associated expression pattern across all age-defined patient subgroups. Although its association was less pronounced than that of *KMT2D*, *RFNG*, and *IGF1*, its consistent expression profile suggests that *CDKN2C* may represent an additional component of the recurrence-associated molecular signature. ATM is a central regulator of the cellular DNA damage response and controls multiple processes, including DNA repair, apoptosis, and cell cycle arrest. Although widely recognized as a tumor suppressor, accumulating evidence suggests that context-dependent ATM signaling may also promote tumor progression and therapeutic resistance, including in breast cancer [59]. The mTOR signaling pathway also plays a critical role in tumor development, cellular metabolism, immune regulation, and aging [60,61]. RPTOR and RICTOR are essential components of the mTOR signaling pathway, serving as defining components of the mTORC1 and mTORC2 complexes, respectively [62,63,64,65]. Previous studies have suggested that RPTOR- and RICTOR-mediated signaling networks may contribute to tumor progression and adverse clinical outcomes [66,67]. In our study, *ATM* and *RPTOR* expression levels were elevated primarily in the recurrent geriatric and postmenopausal non-geriatric groups, whereas *RICTOR* exhibited a more heterogeneous expression profile, with recurrence-associated upregulation mainly in the postmenopausal non-geriatric and premenopausal groups. These findings suggest that DNA damage response and mTOR-related signaling networks are differentially regulated across age-defined patient subgroups. Consistent with these observations, KEGG analysis also identified significant enrichment of the FoxO signaling pathway, which functionally interacts with DNA damage response and mTOR-associated signaling networks.

One of the most notable findings of our study was the significant contribution of transcriptomic markers to ML-based prediction models. Boruta feature selection analysis identified *KMT2D*, *CDKN2C*, *RICTOR*, *IGF1*, *RFNG*, *RPTOR*, and *ATM* among the most important variables for recurrence classification. Notably, *KMT2D* emerged as the feature with the highest importance score, followed by age, NPI, and tumor size. These findings suggest that recurrence risk cannot be explained solely by clinical or molecular factors, but rather by the combined contribution. Furthermore, the observation that several genes exhibiting consistent recurrence-associated expression patterns were also selected by the Boruta algorithm supports the concordance between the biological and computational approaches employed in this study. Collectively, these findings further support the biological relevance of the recurrence-associated transcriptomic signatures identified through age-stratified analyses.

The prominence of *KMT2D* in both the biological analyses and the Boruta feature selection process further suggests that this gene represents a promising prognostic biomarker candidate warranting further experimental and clinical validation. Furthermore, integration of transcriptomic signatures with clinical variables improved predictive performance, with XGBoost showing the best performance among the evaluated models. Nevertheless, additional validation in independent patient cohorts is required before potential clinical application.

Overall, these findings were further supported by KEGG pathway enrichment analysis. Integration of subgroup-specific transcriptomic analyses with ML-based feature selection identified biologically relevant genes with predictive value. Comparison with the clinical-only baseline models further demonstrated that transcriptomic biomarkers provided additional predictive information beyond conventional clinicopathological variables, although the improvement in predictive performance was moderate. Although XGBoost achieved the best predictive performance (AUC = 0.71), the model should be regarded as a promising proof-of-concept framework rather than an immediately applicable clinical decision-support tool. External validation and sensitivity analyses further supported the robustness of the principal transcriptomic biomarkers, although additional validation in larger independent cohorts using comparable clinical endpoints remains necessary before clinical application.

Because the present study was based exclusively on transcriptomic analyses, the biological roles and prognostic significance of the identified genes should be considered hypothesis-generating and require further experimental validation. An important limitation of this study is that the original METABRIC PFS endpoint represents a composite clinical outcome encompassing recurrence, locoregional progression, distant metastasis, second primary cancer, and death from any cause. Accordingly, the identified transcriptomic signatures should be interpreted as reflecting the composite PFS endpoint rather than any individual clinical event, and future studies using more clinically homogeneous endpoints will improve their biological and clinical interpretation. Moreover, although age-related confounding was minimized by performing separate differential expression analyses within biologically defined age-stratified subgroups, other clinicopathological variables, including tumor stage, tumor size, and treatment, were not included as covariates in the differential expression analysis. Therefore, residual confounding cannot be completely excluded, and future multivariable studies will be valuable for evaluating the independent contribution of the identified transcriptomic signatures. Finally, as this study represents a retrospective secondary analysis of a publicly available dataset, the findings should be interpreted with appropriate caution despite the independent external validation of the principal candidate genes. Consistent with our previous OS model, the present findings suggest that integrating transcriptomic signatures with clinical characteristics provides a promising framework for personalized prognostic assessment in Luminal A BC. While our previous study focused on overall survival, the present study extends this integrative approach to recurrence-associated outcomes derived from the original METABRIC PFS endpoint [31].

## 5. Conclusions

In this study, recurrence-associated transcriptomic alterations in Luminal A BC were comprehensively evaluated across age-defined patient subgroups using integrated transcriptomic and machine learning approaches. *KMT2D*, *RFNG*, *IGF1*, and *CDKN2C* exhibited consistent recurrence-associated expression patterns across patient subgroups, whereas *ATM*, *RPTOR*, and *RICTOR* displayed subgroup-dependent expression profiles. Machine learning analyses further supported the prognostic relevance of these biomarkers, with *KMT2D* emerging as the most influential molecular predictor. The integration of transcriptomic biomarkers with clinical variables, including age, NPI, and tumor size, improved recurrence classification and identified biologically relevant molecular features associated with disease progression. Collectively, these findings identify candidate transcriptomic biomarkers for recurrence in Luminal A BC that warrant further investigation. Additional validation in large, independent cohorts using comparable clinical endpoints is required to determine the reproducibility and generalizability of these findings.

## Figures and Tables

**Table 1:** Distribution of patients according to age-defined subgroups and recurrence status.

Age Subgroup	Nonrecurrent, *n* (%)	Recurrent, *n* (%)	Total, *n*
Premenopausal	76 (63.9%)	43 (36.1%)	119
Postmenopausal non-geriatric	148 (60.2%)	98 (39.8%)	246
Geriatric	43 (45.7%)	51 (54.3%)	94
Total	267 (58.2%)	192 (41.8%)	459

Abbreviations: *n*, number of patients. Percentages represent the proportion of recurrent and nonrecurrent patients within each age-defined subgroup.

**Table 2:** Selected differentially expressed genes meeting the criteria of FDR ≤ 0.1 and |log_2_FC| ≥ 2 in age-defined Luminal A breast cancer patient groups.

Gene Name	Log_2_ Fold Change	BH-Adjusted *p*-Value (FDR)	Direction
*RFNG*	3.4298	<0.001	Up
*UGT2B17*	3.3504	<0.001	Up
*ATM*	3.3491	<0.001	Up
*RPTOR*	3.2156	<0.001	Up
*GDF2*	3.1251	<0.001	Up
*NUMBL*	2.9478	<0.001	Up
*KMT2C*	2.8304	<0.001	Up
*IGF1*	2.8135	<0.001	Up
*KMT2D*	2.3912	0.014	Up
*CDKN2C*	2.1967	0.026	Up
*AGMO*	−2.2195	0.021	Down
*HSD3B2*	−2.2235	0.019	Down
*MAP3K10*	−2.2253	0.018	Down
*RICTOR*	−2.7493	<0.001	Down
*SLCO1B3*	−2.7775	<0.001	Down

**Table 3:** Top significantly enriched KEGG pathways identified among upregulated and downregulated differentially expressed genes (DEGs).

Direction	KEGG Pathway	FDR	Gene Count	Pathway Size	Fold Enrichment
Up	Cellular senescence	1.17 × 10^−26^	37	156	1.102076049
Up	Pathways in cancer	1.11 × 10^−24^	58	530	5.084948542
Up	Human papillomavirus infection	1.27 × 10^−20^	43	331	0.603635677
Up	Endocrine resistance	1.37 × 10^−20^	26	95	1.271698565
Up	Notch signaling pathway	1.37 × 10^−20^	22	59	1.732627119
Up	Chronic myeloid leukemia	5.82 × 10^−18^	22	76	1.345065789
Up	MicroRNAs in cancer	1.02 × 10^−17^	29	161	8.369635799
Up	Hepatitis B	1.07 × 10^−17^	29	162	0.831797138
Up	Human T-cell leukemia virus 1 infection	8.89 × 10^−16^	31	222	6.488482801
Up	Pancreatic cancer	1.38 × 10^−15^	20	76	1.222787081
Down	Pathways in cancer	5.52 × 10^−7^	16	530	5.878167116
Down	Pancreatic cancer	9.73 × 10^−6^	7	76	1.793421053
Down	FoxO signaling pathway	1.80 × 10^−5^	8	131	1.189094875
Down	Human papillomavirus infection	2.91 × 10^−5^	11	331	6.470867501
Down	Hepatitis B	5.50 × 10^−5^	8	162	9.615520282
Down	Thyroid hormone signaling pathway	7.90 × 10^−5^	7	121	1.126446281
Down	Colorectal cancer	1.30 × 10^−4^	6	86	1.358471761
Down	Viral carcinogenesis	1.79 × 10^−4^	8	202	0.771145686
Down	Endocrine resistance	1.80 × 10^−4^	6	95	1.229774436
Down	Steroid hormone biosynthesis	3.02 × 10^−4^	5	62	1.570.276.498

**Table 4:** Distribution of molecular subtypes and clinical events in the GSE20685 validation cohort.

Subtype	*n*	Event_Metastasis	Regional_Relapse	Deaths
I	37	6	2	7
II	34	16	5	17
III	41	8	4	9
IV	81	29	5	29
V	41	2	1	3
VI	93	22	8	18

**Table 5:** External validation of the principal candidate genes in the independent GSE20685 cohort using Cox proportional hazards regression analysis for metastasis-free survival.

Endpoint	Gene	Probe	*n*	Events	HR	95% CI	*p*	Logrank_p	Median Expression	Direction
Metastasis-free survival	*KMT2D*	211791_s_at	134	24	0.421	0.179–0.992	0.048	0.042	4.3318	High expression associated with better outcome
Metastasis-free survival	*RFNG*	212969_x_at	134	24	0.481	0.206–0.524	0.038	0.041	9.5963	High expression associated with better outcome
Metastasis-free survival	*IGF1*	209542_x_at	134	24	0.846	0.377–1.899	0.685	0.684	9.0222	High expression associated with worse outcome
Metastasis-free survival	*CDKN2C*	204159_at	134	24	1.086	0.488–2.420	0.839	0.839	9.3127	Low expression associated with worse outcome
Metastasis-free survival	*ATM*	208442_s_at	134	24	0.341	0.099–0.586	0.018	0.012	9.7347	High expression associated with better outcome
Metastasis-free survival	*RICTOR*	226310_at	134	24	1.016	1.001–1.032	0.042	0.039	9.2738	Low expression associated with better outcome
Metastasis-free survival	*RPTOR*	218668_s_at	134	24	1.099	0.492–2.455	0.819	0.819	10,7275	High expression associated with worse outcome

HR, hazard ratio; CI, confidence interval. Hazard ratios compare patients with high versus low gene expression. Statistically significant associations (*p* < 0.05) are indicated in bold.

**Table 6:** Comparison of continuous clinical variables between recurrent and nonrecurrent Luminal A BC patients. Values are presented as mean ± standard deviation. Group comparisons were performed using the independent samples *t*-test. Statistical significance was defined as *p* < 0.05. Effect sizes are reported as Hedges’ g with corresponding 95% CI.

Group ($\bar{X}$ ± SD)	Nonrecurrent (*n* = 267)	Recurrent (*n* = 192)	*p*-Value	Effect Size (Hedges’ g)	95% CI
Age	57.4 ± 11.0	61.1 ± 13.2	<0.001	0.31	0.12 to 0.50
Tumor Size	21.7 ± 9.7	26.8 ± 15.2	<0.001	0.41	0.23 to 0.60
Months	151.1 ± 71.8	85.5 ± 64.6	<0.001	−0.95	−1.15 to −0.76
Nottingham Prognostic Index	3.4 ± 1.0	3.9 ± 1.2	<0.001	0.46	0.27 to 0.65

**Table 7:** Association between categorical clinicopathological variables and recurrence status in Luminal A BC patients. Values are presented as counts and percentages. *p*-values were obtained using the chi-square test. Cramer’s V is reported as the effect size measure.

Group (Count (%))	Nonrecurrent (*n* = 267)	Recurrent (*n* = 192)	*p*-Value *	Effect Size (Cramer’s V)
**Type of breast surgery**				
Mastectomy	128 (48%)	122 (64%)	**<0.001**	0.157
Breast Conserving Surgery	139 (52%)	70(36%)		
**Chemotherapy**				
Received	26 (10%)	25 (13%)	0.27	0.052
Not received	241 (90%)	167 (87%)		
**Radiotherapy**				
Received	168 (63%)	100 (52%)	**0.02**	0.108
Not received	99 (37%)	92 (48%)		
**Histological grade**				
Low	60 (22%)	23 (12%)	**<0.001**	
Moderate	149 (56%)	106 (55%)		0.174
High	58 (22%)	63 (33%)		
**Histopathological type**				
Invasive Ductal Carcinoma (IDC)	200 (75%)	140 (73%)	**0.024**	
Invasive Lobular Carcinoma (ILC)	31 (12%)	16 (8%)		0.093
Mixed Tumor IDC + ILC	36 (13%)	36 (19%)		
**Primary Tumor Laterality**				
Right	138 (52%)	86 (45%)	0.216	0.058
Left	129 (48%)	106 (55%)		
**Cellularity**				
High	124 (46%)	88 (46%)	0.737	
Moderate	120 (45%)	91 (47%)		0.036
Low	23 (9%)	13 (7%)		
**HER 2 loss snp 6**				
Yes	8 (3%)	7 (4%)	0.699	0.018
No	259 (97%)	185 (96%)		
**Tumor stage**				
Stage 1	121 (45%)	62 (32%)	**0.002**	
Stage 2	119 (45%)	98 (51%)		0.176
Stage 3	27 (10%)	22 (11%)		
Stage 4	0 (0%)	10 (5%)		

* Indicates statistical significance (*p* < 0.05); significant results are shown in bold.

**Table 8:** Hyperparameter optimization space used for ML model development.

Model	Parameter	Values
RF	mtry	2, 3, 4, 5, 6
	nodesize	1, 3, 5
	ntree	50, 100, 150, 200, 250
MLP	size	5, 10, 15, 20
	decay	0, 0.001, 0.01
	maxit	100, 200, 500
LR	family	binomial
	link function	logit
	regularization	none
XGB	nrounds	100, 200, 300, 400, 500
	eta	0.01, 0.05, 0.1
	gamma	0, 1, 3, 5
	colsample_bytree	0.4, 0.5, 0.6
	max_depth	3, 4, 5, 6
	Subsample	0.6, 0.7, 0.8
	min_child_weight	1, 3, 5, 7

**Table 9:** Performance metrics of ML classifiers in training and independent testing cohorts for recurrence classification in Luminal A BC patients.

Classifiers	Diagnostic Accuracy	Sensitivity	Specificity	F1 Score	Positive Predictive Value	Negative Predictive Value	AUC
**MLP**							
Training	0.69	0.64	0.71	0.7	0.78	0.56	0.58
95%CI	(0.62–0.72)	(0.57–0.71)	(0.63–0.78)	(0.65–0.75)	(0.73–0.83)	(0.51–0.61)	(0.52–0.64)
Testing	0.68	0.71	0.64	0.73	0.75	0.59	0.60
95%CI	(0.58–0.75)	(0.61–0.77)	(0.58–0.72)	(0.65–0.80)	(0.65–0.83)	(0.51–0.67)	(0.54–0.66)
**LR**							
Training	0.71	0.68	0.75	0.74	0.81	0.61	0.68
95%CI	(0.66–0.76)	(0.61–0.74)	(0.68–0.81)	(0.69–0.78)	(0.75–0.86)	(0.53–0.68)	(0.62–0.74)
Testing	0.73	0.75	0.71	0.77	0.8	0.65	0.71
95%CI	(0.65–0.80)	(0.67–0.83)	(0.65–0.77)	(0.70–0.84)	(0.72–0.87)	(0.58–0.72)	(0.65–0.77)
**RF**							
Training	0.76	0.74	0.79	0.79	0.84	0.66	0.69
95%CI	(0.71–0.80)	(0.67–0.80)	(0.72–0.85)	(0.75–0.83)	(0.79–0.89)	(0.58–0.73)	(0.63–0.75)
Testing	0.76	0.76	0.77	0.8	0.84	0.66	0.70
95%CI	(0.70–0.82)	(0.68–0.89)	(0.70–0.84)	(0.73–0.86)	(0.75–0.92)	(0.59–0.73)	(0.64–0.76)
**XGBoost**							
Training	0.80	0.81	0.8	0.84	0.87	0.73	0.72
95%CI	(0.76–0.85)	(0.75–0.86)	(0.73–0.85)	(0.80–0.87)	(0.82–0.91)	(0.65–0.80)	(0.67–0.77)
Testing	0.78	0.82	0.73	0.83	0.84	0.71	0.71
95%CI	(0.71–0.85)	(0.76–0.88)	(0.68–0.79)	(0.76–0.88)	(0.77–0.91)	(0.65–0.77)	(0.66–0.76)

## Data Availability

The data are available from the authors on reasonable request.

## Supplementary Materials

The following supporting information can be downloaded at: https://www.mdpi.com/article/10.3390/biology15141160/s1, Table S1: Sensitivity analysis of differentially expressed genes using a more stringent Benjamini–Hochberg adjusted false discovery rate (FDR ≤ 0.05); Table S2: Performance metrics of machine learning classifiers using only clinicopathological variables for binary PFS event classification in Luminal A breast cancer patients. The clinical-only models were developed using age at diagnosis, tumor size, Nottingham Prognostic Index (NPI), type of breast surgery, radiotherapy status, histological grade, histopathological subtype, and tumor stage. The dataset was randomly divided into training (70%) and independent testing (30%) cohorts, and model development was performed using stratified 5-fold cross-validation. Performance is reported as accuracy, sensitivity, specificity, F1-score, positive predictive value (PPV), negative predictive value (NPV), and area under the receiver operating characteristic curve (AUC). Values in parentheses indicate 95% confidence intervals estimated by bootstrap resampling; Table S3: Bootstrap stability analysis of Boruta-selected transcriptomic and clinical features based on 100 bootstrap resamples.
