# Supplementary material for: Age-Specific Transcriptomic Signatures for Classification of Progression-Free Survival Outcomes in Luminal A Breast Cancer: An Integrative Machine Learning Approach
Source: Biology (Basel). 2026 Jul 15;15(14):1160. doi: 10.3390/biology15141160 (PMC13405475; doi:10.3390/biology15141160)
Supplement: Supplementary file 1 [file biology-15-01160-s001.zip › biology-4429551-supplementary.pdf]

Table S1. Sensitivity analysis of differentially expressed genes using a more stringent Benjamini–Hochberg adjusted false discovery rate (FDR ≤ 0.05).

Gene	log2FC	BH-adjusted FDR	Significant (FDR≤0.10)	Significant (FDR≤0.05)
RFNG	3.4298	<0.001	Yes	Yes
UGT2B17	3.3504	<0.001	Yes	Yes
ATM	3.3491	<0.001	Yes	Yes
RPTOR	3.2156	<0.001	Yes	Yes
GDF2	3.1251	<0.001	Yes	Yes
NUMBL	2.9478	<0.001	Yes	Yes
KMT2C	2.8304	<0.001	Yes	Yes
IGF1	2.8135	<0.001	Yes	Yes
KMT2D	2.3912	0.014	Yes	Yes
CDKN2C	2.1967	0.026	Yes	Yes
AGMO	-2.2195	0.021	Yes	Yes
HSD3B2	-2.2235	0.019	Yes	Yes
MAP3K10	-2.2253	0.018	Yes	Yes
RICTOR	-2.7493	<0.001	Yes	Yes
SLCO1B3	-2.7775	<0.001	Yes	Yes

Table S2. Performance metrics of machine learning classifiers using only clinicopathological variables for binary PFS event classification in Luminal A breast cancer patients. The clinical-only models were developed using age at diagnosis, tumor size, Nottingham Prognostic Index (NPI), type of breast surgery, radiotherapy status, histological grade, histopathological subtype, and tumor stage. The dataset was randomly divided into training (70%) and independent testing (30%) cohorts, and model development was performed using stratified 5-fold cross-validation. Performance is reported as accuracy, sensitivity, specificity, F1-score, positive predictive value (PPV), negative predictive value (NPV), and area under the receiver operating characteristic curve (AUC). Values in parentheses indicate 95% confidence intervals estimated by bootstrap resampling.

Classifier	Cohort	N	TN	FP	FN	TP	Accuracy (95% CI)	Sensitivity (95% CI)	Specificity (95% CI)	F1 Score (95% CI)	Positive Predictive Value (95% CI)	Negative Predictive Value (95% CI)	AUC (95% CI)
MLP	Training	320	185	1	134	0	0.579 (0.526–0.632)	Nan (-)	0.995 (0.983–1.000)	Nan (-)	Nan (-)	0.581 (0.527–0.637)	0.457 (0.396–0.525)
MLP	Testing	139	79	2	56	2	0.587 (0.514–0.674)	0.034 (0.000–0.092)	0.988 (0.961–1.000)	0.066 (0.000–0.164)	0.667 (0.000–1.000)	0.585 (0.513–0.668)	0.570 (0.482–0.660)
LR	Training	320	149	38	63	70	0.682 (0.632–0.732)	0.522 (0.442–0.610)	0.797 (0.741–0.853)	0.579 (0.505–0.651)	0.648 (0.555–0.740)	0.700 (0.639–0.755)	0.707 (0.647–0.765)
LR	Testing	139	62	19	31	27	0.645 (0.561–0.721)	0.466 (0.330–0.582)	0.775 (0.695–0.873)	0.524 (0.400–0.625)	0.600 (0.461–0.750)	0.667 (0.569–0.765)	0.674 (0.574–0.756)
RF	Training	320	147	43	74	56	0.633 (0.532–0.716)	0.332 (0.375–0.529)	0.778 (0.689–0.863)	0.489 (0.391–0.634)	0.656 (0.470–0.719)	0.665 (0.590–0.797)	0.691 (0.563–0.730)
RF	Testing	139	64	17	35	23	0.630 (0.551–0.717)	0.397 (0.275–0.536)	0.800 (0.704–0.884)	0.474 (0.344–0.604)	0.590 (0.428–0.737)	0.646 (0.547–0.742)	0.641 (0.537–0.737)
XGB	Training	320	150	36	64	70	0.688 (0.566–0.728)	0.521 (0.446–0.684)	0.801 (0.724–0.883)	0.583 (0.427–0.614)	0.661 (0.584–0.772)	0.712 (0.632–0.776)	0.668 (0.551–0.782)
XGB	Testing	139	64	17	35	23	0.630 (0.558–0.710)	0.397 (0.291–0.517)	0.800 (0.704–0.884)	0.474 (0.361–0.588)	0.590 (0.438–0.750)	0.646 (0.558–0.730)	0.622 (0.527–0.715)

Table S3. Bootstrap stability analysis of Boruta-selected transcriptomic and clinical features based on 100 bootstrap resamples.

Feature	Selection frequency (%)	Mean RF importance	SD RF importance	Number of encoded columns	Stability category
nottingham_prognostic_index	98	0,05513	0,01186	1	High (≥80%)
age_at_diagnosis	92	0,04855	0,01442	1	High (≥80%)
tumor_size	85	0,04443	0,01502	1	High (≥80%)
kmt2d	80	0,03715	0,00964	1	High (≥80%)
kmt2c	65	0,03128	0,00788	1	Moderate (50–79%)
rictor	63	0,03162	0,00709	1	Moderate (50–79%)
hsd3b2	60	0,03151	0,00879	1	Moderate (50–79%)
igf1	60	0,03017	0,00763	1	Moderate (50–79%)
rptor	59	0,02925	0,00823	1	Moderate (50–79%)
gdf2	56	0,02987	0,00737	1	Moderate (50–79%)
slco1b3	56	0,02915	0,00709	1	Moderate (50–79%)
cdkn2c	55	0,02854	0,00789	1	Moderate (50–79%)
ugt2b17	55	0,02813	0,00669	1	Moderate (50–79%)
agmo	53	0,02769	0,00744	1	Moderate (50–79%)
map3k10	53	0,02702	0,00714	1	Moderate (50–79%)
atm	53	0,0271	0,00789	1	Moderate (50–79%)
rfng	51	0,02578	0,00629	1	Moderate (50–79%)
numbl	50	0,02495	0,0061	1	Moderate (50–79%)
type_of_breast_surgery	3	0,01486	0,00964	2	Low (<50%)
neoplasm_histologic_grade	1	0,01118	0,00556	1	Low (<50%)
tumor_other_histologic_subtype	0	0,00918	0,00387	6	Low (<50%)
cellularity	0	0,00852	0,00311	3	Low (<50%)

tumor_stage	0	0,00667	0,00443	1	Low (<50%)
primary_tumor_laterality	0	0,00667	0,00339	2	Low (<50%)
radio_therapy	0	0,00591	0,00387	1	Low (<50%)
chemotherapy	0	0,00157	0,0014	1	Low (<50%)

Selection frequency (%) represents the percentage of bootstrap resamples in which each feature was selected by the Boruta algorithm. Mean RF importance and SD RF importance denote the mean and standard deviation of Random Forest variable importance across bootstrap iterations. Stability categories were defined as High (≥80%), Moderate (50–79%), and Low (<50%) according to feature selection frequency.
